# Dwell Time Outperforms Social and Chemical Predictors of Behavioural Transitions in Ants

**DOI:** 10.3390/e28040451

**Published:** 2026-04-15

**Authors:** Michael Crosscombe, Ilya Horiguchi, Shigeto Dobata, Takashi Ikegami

**Affiliations:** 1 Department of General Systems Studies, Graduate School of Arts and Sciences, The University of Tokyo, Tokyo 153-8902, Japan; ilya-horiguchi@g.ecc.u-tokyo.ac.jp (I.H.); dobata@g.ecc.u-tokyo.ac.jp (S.D.); ikeg@sacral.c.u-tokyo.ac.jp (T.I.); 2 RIKEN Center for Brain Science, Wako 351-0198, Japan; 3 Alternative Machine Inc., Tokyo 153-0041, Japan

**Keywords:** collective behaviour, ant clustering, discrete-time hazard models, behavioural inertia, dwell time, stigmergy, model comparison, *Pristomyrmex punctatus*

## Abstract

Agent-based models of collective behaviour can reproduce the macroscopic patterns observed in biological systems, yet reproducing observed behaviour does not guarantee the model captures the true underlying mechanisms. In ant colonies, for example, clustering may arise from local imitation, chemical marking of the environment, or internal physiological states. Distinguishing between these requires predictive tests at the individual level. Here, we apply regularised hazard models to trajectory data from three colonies and systematically compare candidate mechanisms. We find that neighbour-based cues alone are weak predictors of when an ant will transition between moving and resting states. A reconstructed arrestant pheromone field is similarly weak as a predictor, and combining pheromone with neighbour cues yields inconsistent results across colonies. In contrast, a simple measure of internal state, i.e., how long an ant has occupied its current state, emerges as the dominant predictor. These results suggest that the timing of behavioural transitions is primarily governed by internal dynamics, while environmental and social cues act as modulators that shape where transitions occur rather than when.

## 1. Introduction

Agent-based models have been largely successful at reproducing the collective patterns observed in biological systems. In social insects, for example, simple local rules can generate complex collective behaviours such as clustering, decision-making, and coordinated foraging, which have been reproduced in simulation [1,2,3,4]. But many different microscopic rules can produce the same macroscopic outcome, so matching a simulation to data is not sufficient to prove that a model accurately captures a particular mechanism [5,6]. To this end, we instead seek to identify which subset of features are most predictive of individual behaviour.

Clustering in ant colonies provides a useful test case. When ants aggregate into dense, static groups, at least three distinct mechanisms could be responsible. First, local imitation: an ant may be more likely to stop moving when surrounded by resting neighbours, and more likely to resume movement when nearby ants depart [4,7]. Second, environmental memory: social insects deposit chemical signals that persist in the substrate, and such pheromone fields could bias subsequent visitors towards stopping in marked areas. Arrestant pheromones that induce aggregation have been chemically identified in termites [8], and analogous signals likely operate in ants. Third, internal state: an ant’s decision to transition may depend primarily on how long it has already been moving or resting, reflecting fatigue, satiation, or some other physiological variable rather than external cues. Individual ants exhibit intrinsic activity-rest cycles [9], response thresholds that vary independently of the environment [10], and hormonal states that regulate behavioural transitions on longer timescales [11].

These hypotheses are not mutually exclusive, but they differ in where information is stored. Local imitation treats the environment as static and decisions as instantaneous reactions to neighbours. Environmental memory introduces persistence in the medium: the pheromone field encodes the shared history of ant activity. Internal state places memory inside the agent, so that past behaviour influences future transitions even when the external context is unchanged. Distinguishing between these requires predictive tests at the level of individual ants rather than aggregate statistics.

The standard approach in the collective behaviour literature has been to assume that state durations follow an exponential distribution, implying a memoryless Markov process in which the probability of transitioning is constant over time [7]. This assumption is convenient but rarely tested. If ants instead exhibit behavioural inertia (i.e., a tendency to remain in their current state the longer they have occupied it), then statistical models that ignore dwell time will misattribute this regularity to social cues, producing false positives for local imitation.

In this paper, we apply regularised hazard models to trajectory data from three colonies of *Pristomyrmex punctatus* and systematically compare the predictive power of competing mechanisms. We begin with a neighbour-only model that includes local density and the fraction of resting neighbours. We then reconstruct an arrestant pheromone field under an accumulation hypothesis and test whether it provides additional predictive power. Finally, we incorporate dwell time as a proxy for internal state. By evaluating each model using out-of-fold AUC on held-out ants, we show that neighbourhood features alone are weak predictors (AUC 0.53–0.60 for stops, 0.65–0.72 for starts), that the pheromone field provides no consistent additional discrimination when combined with social context, and that dwell time dominates all other features (AUC 0.64–0.72 for stops, 0.73–0.83 for starts). These results suggest that the timing of behavioural transitions is governed primarily by internal dynamics, with environmental and social cues acting as modulators rather than primary drivers.

The remainder of the paper is structured as follows. In Section 2, we review related work on collective behaviour, stigmergy, and survival analysis. In Section 3, we describe the experimental setup and dataset. In Section 4, we introduce the hazard model framework and candidate covariates. In Section 5, we present model comparisons, negative controls, and cross-colony generalisation results. We conclude with a discussion of implications for agent-based modelling.

## 2. Background

Clustering is a canonical example of self-organisation in social insects. Deneubourg et al. [12] showed that simple probabilistic rules, in which ants pick up and deposit items with probabilities that depend on local density, are sufficient to generate stable clusters through positive feedback. Similar density-dependent rules produce brood sorting [13,14]. In both cases, the macroscopic pattern arises from local rules that depend only on the immediate neighbourhood of each ant.

The dominant modelling paradigm treats individuals as self-propelled particles responding to their local neighbourhood [15]. These models reproduce collective patterns, but as Sumpter et al. [16] have noted, most are sufficient rather than necessary: alternative models based on different assumptions often perform equally well. Katz et al. [17] make the point concisely: even if a model matches data across a set of observables, there is no guarantee that it will share the same response to perturbation unless the underlying rules are also correct. Mann et al. [18] showed that Markovian self-propelled particle models can capture fine-scale interaction rules but fail to reproduce global dynamics; non-Markovian models with memory of previous interactions proved necessary to match data at multiple scales. This finding directly motivates our inclusion of dwell time as a covariate.

An alternative to purely local interaction is stigmergy: coordination mediated by modifications to the environment [19]. Theraulaz and Bonabeau [20] distinguished quantitative stigmergy (based on concentration gradients, as in pheromone trails) from qualitative stigmergy (based on spatial configurations, as in nest architecture). Trail pheromones provide the canonical example: ants deposit pheromone as they walk, and subsequent ants preferentially follow paths with higher concentrations [21]. For clustering, the relevant signal is likely an arrestant pheromone that accumulates where individuals rest. Mitaka et al. [8] identified such an arrestant in termites, where non-volatile lipids induce long-term aggregation. Analogous signals likely operate in ants, though chemical identification remains incomplete. If such a pheromone influences stopping probability, it introduces spatial memory: the chemical field encodes where individuals have been, not just where they are now.

A common assumption in models of collective behaviour is that state durations follow an exponential distribution, implying a memoryless process in which transition probability is constant over time. Minasandra et al. [22] demonstrated that behavioural bouts instead exhibit decreasing hazard functions across multiple mammal species: the longer a bout progresses, the less likely it is to end. If ants exhibit similar behavioural inertia, models that ignore dwell time will misattribute this regularity to external cues, producing false positives for local imitation or stigmergy. A related issue is unobserved individual heterogeneity; Vaupel et al. [23] introduced frailty models that accommodate variation in individual propensities through multiplicative random effects on hazards.

Survival analysis provides a natural framework for modelling behavioural state transitions. The Cox proportional hazards model [24] remains foundational; Andersen and Gill [25] extended it to time-varying covariates and recurrent events. For data recorded at discrete intervals, Prentice and Gloeckler [26] showed that grouped survival data with proportional hazards yields a complementary log–log model, while Allison [27] provided the logistic alternative. Singer and Willett’s textbook [28] remains the definitive practical guide for discrete-time hazard models.

Applications to animal behaviour include Asher et al. [29], who demonstrated survival analysis and multistate model applications to behavioural transitions in veterinary contexts. Patterson et al. [30] used Hidden Markov Models to classify movement behaviour in marine animals. However, the application of hazard models to collective behaviour questions (i.e., asking which covariates predict state transitions in social insects) remains underutilised. Our approach fills this gap by fitting logistic hazard models to ant trajectory data and comparing the predictive power of competing mechanisms.

## 3. Experiment

We analyse trajectory data from three colonies of *Pristomyrmex punctatus*, a queenless ant native to Japan in which all workers reproduce clonally by thelytokous parthenogenesis [31,32]. This species nests in pre-existing cavities such as fallen twigs and leaf litter rather than constructing nests, and colonies relocate frequently [33]; aggregation is their primary mode of establishing a nest site. The clonal workforce eliminates genetic variation among nestmates, simplifying individual-level analysis. Each colony was placed in a circular arena (10 cm diameter) constructed from two transparent perspex sheets separated by a gap slightly larger than ant body height, restricting movement to a single layer. No food or brood were present, isolating clustering behaviour from foraging-related aggregation. Colonies were filmed from above for 4 h, with recording beginning immediately after placement. Videos were recorded at 60 frames per second and downsampled to 10 fps for analysis. Individual ants were identified in each frame using a U-Net segmentation model [34] and tracked by positional correspondence between consecutive frames, yielding coordinates (x,y) for each ant at each frame. At our imaging resolution, the scale factor is 0.083 mm/pixel and ant body length is approximately 2.5 mm (30 pixels). Throughout this paper, we express spatial quantities in body lengths (BL), where 1 BL ≈ 2.5 mm.

We classify each ant at each frame as either moving or resting based on instantaneous speed. The speed distribution is bimodal, with a clear separation between resting ants (mean speed 0.26 mm/s) and moving ants (mean speed 5.2 mm/s). We use a threshold of 0.25 mm/s (3 pixels/frame at 10 fps) to assign states, with minimum state durations of 1.0 s for resting and 0.5 s for moving to filter transient fluctuations.

**Figure 1:**
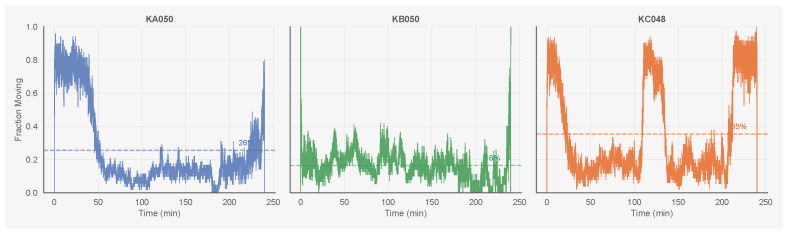
Fraction of ants in the moving state over time for all three colonies. Activity levels and temporal dynamics vary across colonies, with KC048 showing the highest mean activity (35%) and KB050 the lowest (15%).

The three colonies contain 50 (KA050), 50 (KB050), and 48 (KC048) ants, with data validity (proportion of frames with successful tracking) ranging from 79% to 93%. Activity levels vary across colonies: KC048 is most active (35% of time moving), KB050 least active (15%), and KA050 intermediate (25%). The number of state transitions per ant ranges from 324 (KB050) to 673 (KC048), providing sufficient data for hazard model estimation. Figure 1 shows the fraction of ants moving over time; all colonies exhibit fluctuations between periods of high and low collective activity.

**Figure 2:**
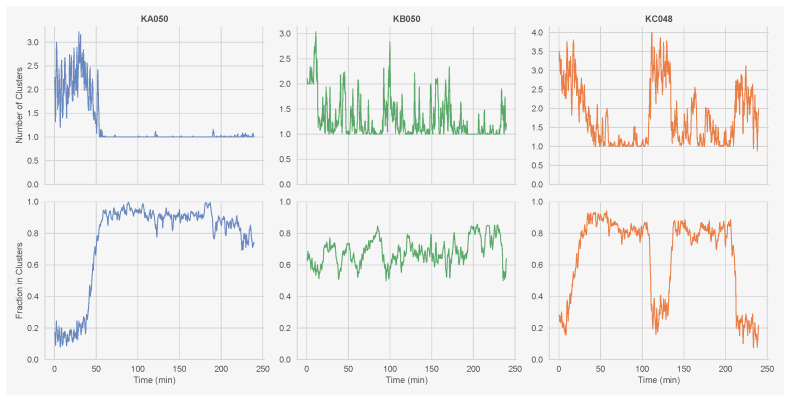
Clustering dynamics over time for all three colonies. Top row: number of spatial clusters. Bottom row: fraction of ants belonging to clusters. Clustering is computed using DBSCAN with ϵ=1.5 BL and a minimum cluster size of 3. Time series are smoothed with a 5 s centred rolling average.

These activity fluctuations produce dynamic clustering patterns. Figure 2 shows the number of spatial clusters and the fraction of ants belonging to clusters over time. Clusters are defined using DBSCAN (density-based spatial clustering of applications with noise) [35] with ϵ=1.5 BL and a minimum cluster size of 3 ants. The number of clusters varies between 2 and 10 across colonies, while the fraction of ants in clusters ranges from 0.4 to 0.9. Understanding what drives individual ants to stop (joining clusters) and start (leaving clusters) is the central question of this paper.

## 4. Model

To distinguish between these mechanisms, we require a statistical framework that can predict individual transitions and attribute predictive power to different covariates. We adopt a discrete-time hazard model: at each video frame, we ask whether an ant will transition in the next frame given its current state and observable context. This framing has two advantages. First, it respects the causal nature of the problem by conditioning only on information available before the transition. Second, it allows us to compare candidate mechanisms by evaluating which covariates improve predictive performance.

### 4.1. Hazard Formulation

Consider an ant *i* observed at discrete time steps t=1,2,… corresponding to video frames recorded at 10 frames per second. At each frame, the ant is in one of two behavioural states: moving or resting. We denote the state by $S_{i}\left(t\right)\in \left\{0,1\right\}$, where $S_{i}\left(t\right)=1$ indicates that the ant is moving and $S_{i}\left(t\right)=0$ indicates that it is resting. A transition occurs when the state changes between consecutive frames, i.e., when $S_{i}\left(t+1\right)\neq S_{i}\left(t\right)$. We refer to a transition from moving to resting as a ‘stop’ and a transition from resting to moving as a ‘start’.

Our goal is to model the probability that an ant will transition in the next frame given its current state and some set of observable covariates. This probability is known as the hazard rate. For stops, we define

(1) $$\lambda_{i}^{\mathrm{stop}}\left(t\right)=P\left(S_{i}\left(t+1\right)=0|S_{i}\left(t\right)=1,\;X_{i,t}\right),$$

where $X_{i,t}$ is a vector of covariates describing the local context of ant *i* at time *t*. An analogous definition holds for starts:

(2) $$\lambda_{i}^{\mathrm{start}}\left(t\right)=P\left(S_{i}\left(t+1\right)=1|S_{i}\left(t\right)=0,\;X_{i,t}\right).$$

We model these hazards using logistic regression, so that

(3) $$\lambda_{i}^{\mathrm{stop}}\left(t\right)=\sigma \left(\beta_{0}+\beta^{⊤}X_{i,t}\right),$$

where $\sigma \left(z\right)=1/\left(1+e^{-z}\right)$ is the logistic function and β is a vector of coefficients to be estimated from data.

Covariates measured at the moment of transition may already reflect the transition itself rather than cause it. For example, an ant that is about to stop may already be decelerating, which would inflate the apparent predictive power of speed-based features. To avoid this leakage, we evaluate all covariates at a fixed lag *k* before the transition frame. In all analyses, we use k=3 frames (0.3 s at 10 fps), which separates the predictor window from the transition event while remaining within the timescale of local interactions.

### 4.2. Candidate Covariates

We consider three families of covariates corresponding to the three candidate mechanisms introduced above.

The simplest hypothesis is that an ant’s transition probability depends only on its immediate neighbours. We compute four features within a fixed radius R=1.5 BL (45 pixels, approximately 3.75 mm): (i) *local density*, the count of neighbours divided by the neighbourhood area $\pi R^{2}$; (ii) *fraction resting*, the proportion of neighbours currently in the resting state; (iii) *speed difference*, the focal ant’s speed minus the mean speed of its neighbours; and (iv) *heading dispersion*, the circular standard deviation of heading differences between the focal ant and its neighbours. These features capture both the spatial configuration and kinematic state of the local neighbourhood. Under the local imitation hypothesis, we expect ants to be more likely to stop when surrounded by resting neighbours and more likely to start when surrounded by moving neighbours. Note that we deliberately exclude cluster membership as a covariate: clusters are an emergent population-level property defined by DBSCAN, and including them would conflate the outcome we seek to explain (why ants stop and form clusters) with a predictor derived from that outcome.

The second hypothesis is that ants deposit a chemical signal (an arrestant pheromone) that accumulates in the substrate and influences subsequent visitors. We reconstruct a pheromone field ϕ(x,t): each ant continuously deposits pheromone at its current position, which diffuses spatially and decays exponentially. Deposition occurs via a Gaussian kernel with radius 0.25 BL, diffusion follows $D=\ell^{2}/\tau$ (coupling spatial and temporal scales), and decay is exponential with time constant τ. The deposition and decay rates are set equal to (1/τ), so that each ant contributes unit integrated mass at steady state.

**Figure 3:**
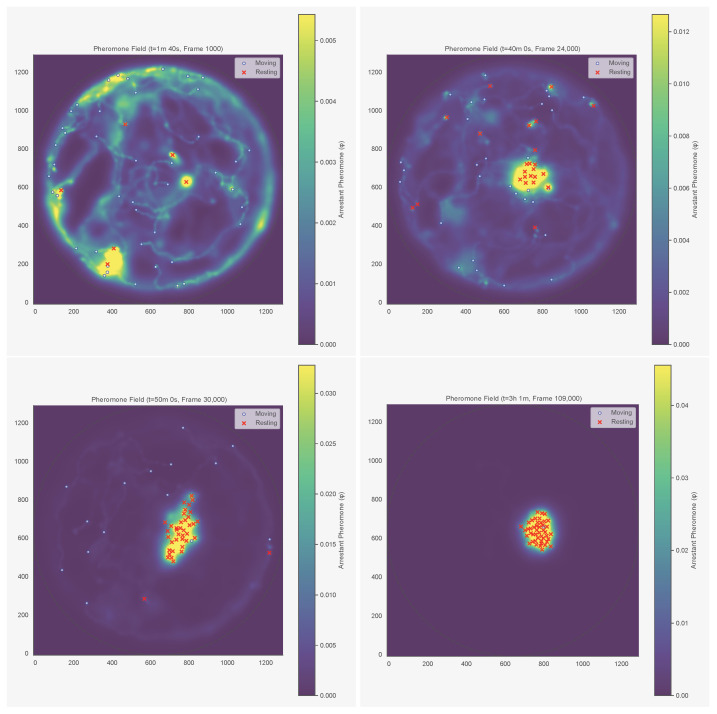
Reconstructed pheromone field at four timepoints (colony KA050). Each panel shows the spatial distribution of accumulated pheromone concentration, with warmer colours indicating higher concentrations. (**Top left**): t=1 min 40 s (early, before aggregation); (**top right**): t=40 min (before aggregation, fraction in clusters ≈0.31; cf. Figure 2); (**bottom left**): t=50 min (mid-aggregation, fraction in clusters ≈0.74); (**bottom right**): t=3 h 1 min (late steady state). Red crosses mark resting ants; white circles mark moving ants. The field accumulates preferentially in areas where ants have spent time resting.

**Figure 4:**
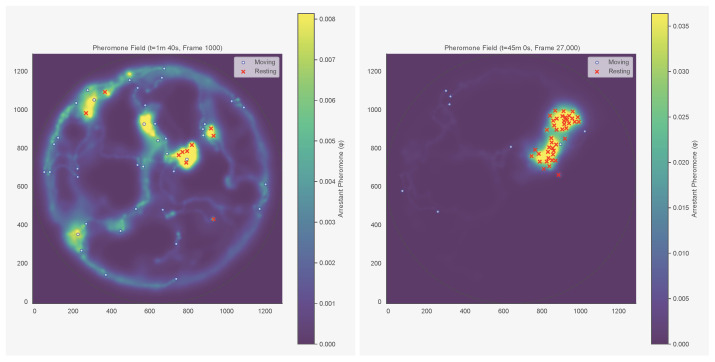

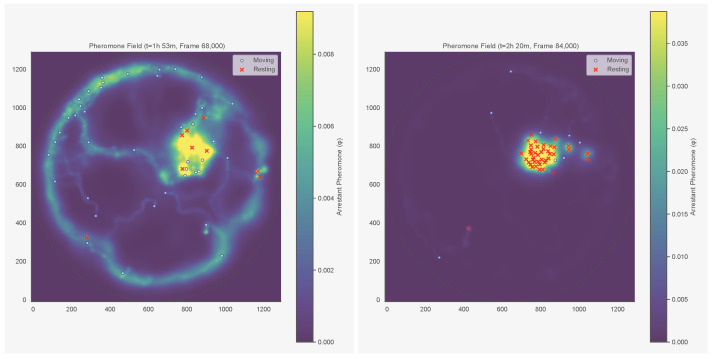
Reconstructed pheromone field at four timepoints (colony KC048), chosen to capture the aggregate–split–reaggregate dynamics visible in Figure 2. (**Top left**): t=1 min 40 s (early, before aggregation); (**top right**): t=45 min (peak of first aggregation); (**bottom left**): t=113 min (during cluster dissolution, fraction in clusters drops to 0.32); (**bottom right**): t=140 min (after reaggregation). The pheromone field retains a spatial memory of the first cluster location even after the colony disperses.

We tested τ∈{60,180,300,600,900,3600} s and ℓ∈{0.5,1.0,1.5,2.0,2.5} BL. The upper bound of 3600 s (1 h) is conservative: in termites, arrestant pheromones remain active for at least 4 h [8]. To isolate spatial memory from instantaneous crowding, we residualised the pheromone signal against local density. For each ant *i* at time *t*, we read off the local pheromone concentration $\phi (x_{i},t)$ and include it as a covariate. Figure 3 shows the reconstructed field at four timepoints for colony KA050, and Figure 4 shows the corresponding field for KC048, whose aggregate–split–reaggregate dynamics are visible in Figure 2.

**Figure 5:**
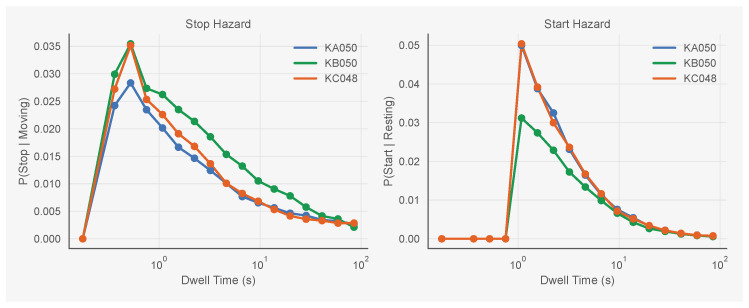
Empirical hazard curves for stop (**left**) and start (**right**) transitions as a function of dwell time. All three colonies show a brief rise after state entry followed by a sustained decline, indicating behavioural inertia. KB050 (green) shows lower hazards overall; KC048 (orange) shows the highest start hazards.

The third hypothesis is that an ant’s transition probability depends on how long it has already occupied its current state. We call this the *dwell time* and denote it by $d_{i}\left(t\right)$. For example, if ant *i* started resting at frame 100 and is still resting at frame 150, then $d_{i}\left(150\right)=50$ frames (5 s). Dwell time enters the model on a log scale: we use $\log(d_{i}\left(t\right)+1)$ as the covariate, which captures the empirical observation that hazard decreases approximately log-linearly with time in state (Figure 5). Including dwell time allows the model to capture behavioural inertia: the tendency for ants to become less likely to transition the longer they have remained in a given state.

### 4.3. Evaluation

To compare these mechanisms, we fit separate logistic hazard models using different subsets of covariates: (i) neighbour features only (local density, fraction resting, speed difference, and heading dispersion), (ii) stigmergy only (pheromone field ϕ), (iii) dwell time only, (iv) neighbour + stigmergy, (v) neighbour + dwell time, and (vi) the full model including all features. We evaluate each model using out-of-fold AUC (area under the receiver operating characteristic curve) under grouped cross-validation, where folds are stratified by ant identity to ensure that the same ant never appears in both training and test sets. AUC measures discrimination: 0.5 indicates chance-level prediction, while 1.0 indicates perfect discrimination. This design respects the within-ant dependence structure and provides an honest estimate of how well each model generalises to held-out individuals.

We use Elastic Net regularisation to stabilise coefficient estimates; the mixing parameter and regularisation strength are tuned by inner cross-validation. Because transitions are rare (occurring in fewer than 1% of at-risk frames), we use incidence-density sampling to construct balanced training sets: for each observed transition, we sample a fixed number of control frames from ants in the same state at the same time who did not transition. This case-control design inflates the apparent event rate but does not affect AUC.

To verify that the observed predictive power is not an artefact of spatial or temporal structure in the data, we implement three negative controls. In the identity shuffle control, we permute ant identities within each frame while keeping positions fixed, then recompute features and refit the model. This removes the link between an ant’s identity and its behavioural history while preserving the overall spatial configuration. In the feature jitter control, we add Gaussian noise (σ equal to each feature’s standard deviation) to the four neighbourhood covariates before refitting the model. If the model relies on genuine covariate values rather than structural correlations, its AUC should degrade under this perturbation. In the dwell-time shuffle control, we permute dwell times across ants within each frame while keeping positions and neighbourhood features fixed, then refit the dwell-only model. This preserves the marginal distribution of dwell times but destroys the correspondence between each ant and its time-in-state. We report permutation *p*-values comparing baseline AUC to the distribution of AUCs under the null. Table 1 summarises the modelling parameters used throughout.

## 5. Results

### 5.1. Empirical Hazard Structure

Figure 5 shows the empirical hazard curves for stop and start transitions across all three colonies. For both transition types, the hazard rises briefly after state entry, peaks around 0.5–1 s, then declines over the following seconds. This pattern holds across all three colonies despite differences in overall activity levels. KB050 (green) shows the lowest hazards overall, matching its low activity level (15% moving), while KC048 (orange) shows the highest start hazards, matching its higher activity level (35% moving).

### 5.2. Model Comparison

**Figure 6:**
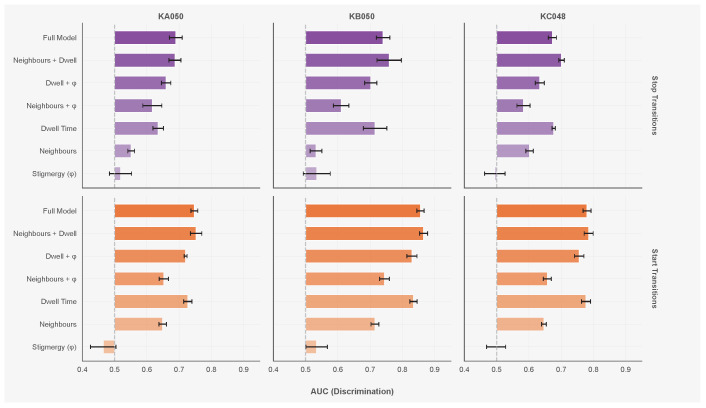
Model comparison across colonies and transition types. Top row: stop transitions; bottom row: start transitions. Columns from left to right: KA050, KB050, KC048. Bars show mean out-of-fold AUC; error bars indicate standard error.

Table 2 reports the out-of-fold AUC for each model variant across all three colonies. Figure 6 shows the comparison visually.

The neighbour-only model achieves an AUC of 0.53–0.60 for stops and 0.65–0.72 for starts across colonies (Table 2). KB050 shows the weakest neighbour effects (stop AUC 0.532), while KC048 shows the strongest (stop AUC 0.602).

The reconstructed pheromone field performs near chance level across all colonies (stop AUC 0.49–0.53, start AUC 0.47–0.53). No parameter combination in the grid search yielded deviance improvements above 0.3%. Adding stigmergy to other models does not improve performance: Neighbours + ϕ achieves lower AUC than Neighbours alone in two of three colonies, and Dwell + ϕ matches or underperforms Dwell alone.

Dwell time alone achieves AUC of 0.64–0.72 for stops and 0.73–0.83 for starts. KB050 shows the strongest duration-dependence (stop AUC 0.716, start AUC 0.834); this colony also has the lowest activity level (15% of time moving). Combining dwell time with neighbourhood features yields an AUC of 0.69–0.76 for stops and 0.75–0.87 for starts. The Neighbours + Dwell model outperforms both single-feature models in all colonies, so duration and neighbour effects provide complementary information. The Full Model (Neighbours + Dwell + ϕ) shows no improvement over Neighbours + Dwell; stigmergy adds no predictive power beyond the other covariates.

### 5.3. Negative Controls

**Figure 7:**
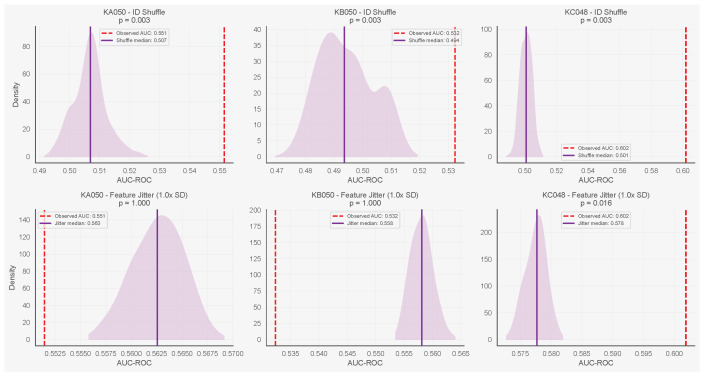
Negative controls for stop transitions (neighbourhood-only model) across all three colonies. Columns: KA050, KB050, KC048. Top row: identity shuffle reduces AUC to chance. Bottom row: feature jitter (1.0× SD) shows modest degradation. Purple shaded regions show kernel density estimates of the null AUC distribution. Dashed vertical lines mark the observed baseline AUC; solid vertical lines mark the null median.

**Figure 8:**
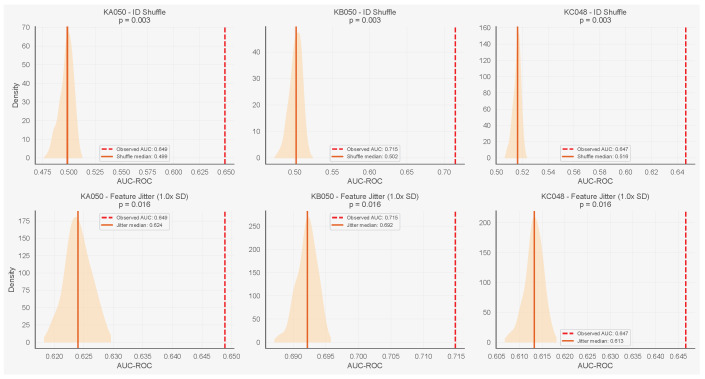
Negative controls for start transitions (neighbourhood-only model) across all three colonies. Columns: KA050, KB050, KC048. Top row: identity shuffle reduces AUC to chance. Bottom row: feature jitter (1.0× SD) degrades AUC, confirming dependence on covariate values. Orange shaded regions show kernel density estimates of the null AUC distribution. Dashed vertical lines mark the observed baseline AUC; solid vertical lines mark the null median.

We first applied two negative controls to validate the neighbourhood features specifically, since neither affects dwell time (which is computed from each ant’s own trajectory). We then applied a third control targeting dwell time directly. In the identity shuffle control, we permuted ant identities within each frame while keeping positions fixed, destroying the correspondence between a focal ant and its neighbours. In the feature jitter control, we added Gaussian noise (σ equal to each feature’s standard deviation) to the four neighbourhood covariates before refitting. Using the neighbourhood-only model, identity shuffle reduced AUC to near chance across all three colonies (median 0.49–0.52), while the observed AUC (0.53–0.65) exceeded the shuffle distribution (p=0.003 in all cases). Feature jitter degraded AUC for start transitions (medians 0.61–0.69 vs. baselines 0.65–0.71, p=0.016), confirming that the model depends on specific feature values. For stop transitions, feature jitter did not degrade AUC; in two of three colonies, the jittered median exceeded the observed value. This is consistent with the weak baseline neighbourhood signal for stops (AUC 0.53–0.60), where the added noise may act more as a regulariser. Figure 7 and Figure 8 show results for all three colonies.

To validate the dwell-time signal itself, we applied an analogous permutation: shuffling dwell times across ants within each frame while keeping positions and neighbourhood features fixed. This preserves the marginal distribution of dwell times in the population but destroys the correspondence between each ant and its time-in-state. Using the dwell-only model, the shuffled AUC dropped to near chance in all colonies and for both transition types (Table 3). In no case did any of the 300 shuffled iterations match or exceed the observed AUC (p=0.003 in all cases, the minimum attainable with 300 permutations and plus-one smoothing). The predictive power of dwell time arises from the specific ant-to-dwell-time mapping, not from structural properties of the dwell-time distribution.

### 5.4. Cross-Colony Generalisation

To assess whether the identified mechanisms generalise across colonies, we trained models on each colony and evaluated predictions on all three (Table 4). For dwell-time-only models, the AUC on each test colony was invariant to the training colony: any colony’s data suffices to learn the negative dwell-time hazard relationship. The combined model (dwell + neighbours) showed minimal cross-colony degradation, with a within-colony AUC of 0.726 and cross-colony AUC of 0.722 for stops. Start transitions showed slightly larger but still modest gaps (within: 0.828, cross: 0.811). The small cross-colony gaps suggest that the learned relationships are not colony-specific.

## 6. Discussion

Dwell time is the strongest single predictor of behavioural state transitions across all three colonies (Table 2). The reconstructed pheromone field performs at chance level, and neighbourhood features, while above chance, are weaker than dwell time alone. The best-performing model combines dwell time with neighbourhood features; social context modulates transition timing but does not drive it. Models that omit internal state will confound duration-dependent dynamics with social effects.

Neighbourhood features predict start transitions (AUC 0.65–0.72) more accurately than stop transitions (AUC 0.53–0.60). A resting ant surrounded by departing neighbours receives a relatively unambiguous cue, whereas a moving ant approaching a resting cluster encounters simultaneous changes in density, heading, and speed that may partially cancel. The cross-colony generalisation data show a parallel pattern: neighbour-only models have a larger cross-colony gap for starts (0.013) than for stops (0.001), while dwell-time models transfer with zero degradation for both transition types (Table 4).

The null result for the pheromone field does not rule out chemical signalling. We tested decay constants from 60 s to 3600 s and diffusion lengths from 0.5 to 2.5 body lengths; no combination improved predictions beyond chance. Our reconstruction assumed accumulation and exponential decay without empirical calibration of deposition rates or chemical identity. Arrestant pheromones have been identified in termites [8], and analogous signals may operate in ants through mechanisms our model does not capture, such as contact-mediated transfer or non-volatile cuticular compounds. Substrate washing or the transfer of conditioned substrate between arenas would provide a more direct test.

Agent-based models of ant clustering typically use reactive rules (e.g., “stop if local density exceeds a threshold”). Our results indicate that such models require at minimum: (i) duration-dependent transition rates, where the probability of switching state decreases with time spent in that state, and (ii) modest social modulation, where neighbour states shift transition probabilities without dominating them. Our models fit population-level coefficients and do not decompose individual variation; incorporating frailty terms [23] or hierarchical structure would allow agents to differ in their baseline transition propensities.

Our hazard model does not include explicit collision mechanics: an ant forced to stop by physical contact with another ant would appear as a density-mediated stop, captured implicitly by the local density and speed difference covariates rather than by an excluded-volume term. The 2D arena constrains movement to a single layer, which may amplify crowding effects relative to natural 3D nest cavities where ants can climb over one another. However, ants in the interior of a 3D cluster are similarly movement-restricted, so the 2D geometry is unlikely to alter the relative importance of dwell time versus neighbourhood features.

## 7. Conclusions

We applied regularised hazard models to trajectory data from three colonies of *Pristomyrmex punctatus* and compared three candidate mechanisms for behavioural transitions: local imitation, stigmergy, and internal state. Our main contributions are: (1) showing that dwell time (AUC 0.64–0.83) outperforms both neighbourhood features (0.53–0.72) and a reconstructed pheromone field (0.47–0.53) as a predictor of state transitions; (2) demonstrating that combining dwell time with neighbourhood features yields the best discrimination (AUC 0.69–0.87), with the learned relationships transferring across colonies; and (3) providing negative controls that validate both the neighbourhood signal and the dwell-time signal, while confirming the pheromone field contributes no additional predictive power.

Several limitations apply. We did not test a pure accumulation model without decay: given that termite arrestant pheromones remain active for at least 4 h with no observed decay [8], such a model may be more appropriate for our 4 h recordings. The three colonies represent a single species in a controlled laboratory environment without food or brood. The hazard models treat each transition independently, ignoring potential coupling between ants beyond the features we measured.

Future work will focus on pheromone perturbation experiments to test whether chemical signals play a role not captured by our reconstruction. We also plan to use simulation-based inference to estimate posterior distributions over model parameters and to test whether the fitted models reproduce macroscopic dynamics such as cluster formation and activity waves. A complementary direction is to evolve neural network controllers for simulated ants using neuroevolution methods such as NEAT [36] and weight-agnostic neural networks [37]. By selectively providing or withholding subsets of the input features identified in this study (neighbourhood cues, dwell time, pheromone), we can test which combinations of information are sufficient to reproduce collective clustering in a simulation.

## Figures and Tables

**Table 1:** Key modelling parameters.

Parameter	Value	Description
Frame rate	10 fps	After downsampling from 60 fps
Predictor lag	3 frames (0.3 s)	Prevents leakage from imminent transitions
Neighbourhood radius	1.5 BL (3.75 mm)	For computing local density and neighbour states
Case:control ratio	10:1	Incidence-density sampling for class balance
Regularisation	Elastic Net	Mixing parameter and strength tuned by CV
Cross-validation	5 folds	Grouped by ant identity
Identity shuffle	300 permutations	Null distribution for permutation test
Feature jitter	$1.0\times \sigma_{\mathrm{feature}}$	Gaussian noise added to covariates
Dwell-time shuffle	300 permutations	Dwell times permuted across ants per frame

**Table 2:** Out-of-fold AUC for hazard models predicting state transitions. Neighbours includes local density, fraction of resting neighbours, speed difference, and heading dispersion. Stigmergy (ϕ) uses the reconstructed pheromone field residualised against density. Dwell uses time since the last transition. Values in parentheses indicate standard error from cross-validation.

Model	KA050	KB050	KC048
*Stop transitions (moving → resting)*			
Stigmergy (ϕ) only	0.518 (0.034)	0.534 (0.042)	0.494 (0.032)
Neighbours only	0.551 (0.010)	0.532 (0.018)	0.602 (0.012)
Dwell only	0.635 (0.016)	0.716 (0.037)	0.677 (0.005)
Neighbours + ϕ	0.617 (0.029)	0.610 (0.024)	0.583 (0.020)
Dwell + ϕ	0.660 (0.014)	0.702 (0.019)	0.634 (0.014)
**Neighbours + Dwell**	**0.687 (0.018)**	**0.759 (0.038)**	**0.701 (0.009)**
Full Model	0.690 (0.020)	0.740 (0.021)	0.673 (0.013)
*Start transitions (resting → moving)*			
Stigmergy (ϕ) only	0.465 (0.040)	0.534 (0.034)	0.498 (0.030)
Neighbours only	0.649 (0.012)	0.715 (0.012)	0.647 (0.007)
Dwell only	0.727 (0.013)	0.834 (0.011)	0.777 (0.014)
Neighbours + ϕ	0.653 (0.014)	0.744 (0.015)	0.657 (0.013)
Dwell + ϕ	0.720 (0.004)	0.829 (0.016)	0.756 (0.015)
**Neighbours + Dwell**	**0.753 (0.018)**	**0.866 (0.012)**	**0.785 (0.014)**
Full Model	0.747 (0.011)	0.856 (0.011)	0.780 (0.012)

**Table 3:** Dwell-time shuffle control. Dwell times were permuted across ants within each frame (300 iterations), then a dwell-only model was refit. The observed AUC exceeds the entire null distribution in all cases.

Colony	Transition	Observed AUC	Shuffle Median	*p*-Value
KA050	Stop	0.635	0.495	0.003
KA050	Start	0.727	0.493	0.003
KB050	Stop	0.716	0.520	0.003
KB050	Start	0.834	0.507	0.003
KC048	Stop	0.677	0.499	0.003
KC048	Start	0.777	0.511	0.003

**Table 4:** Cross-colony generalisation. Models trained on each colony were evaluated on all three; within-colony AUC is the mean diagonal, cross-colony AUC is the mean off-diagonal. The near-zero gap for dwell-only models indicates that the dwell-time hazard relationship generalises across these colonies.

Model	Within-Colony AUC	Cross-Colony AUC	Gap
*Stop transitions (moving → resting)*			
Dwell only	0.717	0.717	0.000
Neighbour only	0.655	0.654	0.001
Dwell + Neighbour	0.726	0.722	0.005
*Start transitions (resting → moving)*			
Dwell only	0.814	0.814	0.000
Neighbour only	0.788	0.775	0.013
Dwell + Neighbour	0.828	0.811	0.017

## Data Availability

Processed trajectory data, extracted features, and analysis code are available at https://github.com/toohuman/ant-dwell-time, accessed on 14 February 2026. Raw video recordings are available from the corresponding author upon reasonable request.

## Abbreviations

The following abbreviations are used in this manuscript:

AUC	Area under the receiver operating characteristic curve
BL	Body length
CV	Cross-validation
DBSCAN	Density-based spatial clustering of applications with noise
fps	Frames per second
